# Global Gene Expression Profiling in PAI-1 Knockout Murine Heart and Kidney: Molecular Basis of Cardiac-Selective Fibrosis

**DOI:** 10.1371/journal.pone.0063825

**Published:** 2013-05-28

**Authors:** Asish K. Ghosh, Sheila B. Murphy, Raj Kishore, Douglas E. Vaughan

**Affiliations:** Feinberg Cardiovascular Research Institute, Feinberg School of Medicine, Northwestern University, Chicago, Illinois, United States of America; Albert Einstein College of Medicine, United States of America

## Abstract

Fibrosis is defined as an abnormal matrix remodeling due to excessive synthesis and accumulation of extracellular matrix proteins in tissues during wound healing or in response to chemical, mechanical and immunological stresses. At present, there is no effective therapy for organ fibrosis. Previous studies demonstrated that aged plasminogen activator inhibitor-1(PAI-1) knockout mice develop spontaneously cardiac-selective fibrosis without affecting any other organs. We hypothesized that differential expressions of profibrotic and antifibrotic genes in PAI-1 knockout hearts and unaffected organs lead to cardiac selective fibrosis. In order to address this prediction, we have used a genome-wide gene expression profiling of transcripts derived from aged PAI-1 knockout hearts and kidneys. The variations of global gene expression profiling were compared within four groups: wildtype heart vs. knockout heart; wildtype kidney vs. knockout kidney; knockout heart vs. knockout kidney and wildtype heart vs. wildtype kidney. Analysis of illumina-based microarray data revealed that several genes involved in different biological processes such as immune system processing, response to stress, cytokine signaling, cell proliferation, adhesion, migration, matrix organization and transcriptional regulation were affected in hearts and kidneys by the absence of PAI-1, a potent inhibitor of urokinase and tissue-type plasminogen activator. Importantly, the expressions of a number of genes, involved in profibrotic pathways including Ankrd1, Pi16, Egr1, Scx, Timp1, Timp2, Klf6, Loxl1 and Klotho, were deregulated in PAI-1 knockout hearts compared to wildtype hearts and PAI-1 knockout kidneys. While the levels of Ankrd1, Pi16 and Timp1 proteins were elevated during EndMT, the level of Timp4 protein was decreased. To our knowledge, this is the first comprehensive report on the influence of PAI-1 on global gene expression profiling in the heart and kidney and its implication in fibrogenesis and several other biological processes. The significance of these observations in the light of heart-specific profibrotic signaling and fibrogenesis are discussed.

## Introduction

In response to injury, the body’s defense system immediately responds leading to induction of inflammation which is an essential event during wound healing. Initially, vascular injury causes mononuclear cell infiltration/inflammation in affected organs. The infiltrating cells produce a wide variety of cytokines including the profibrotic cytokine TGF-ß that activates fibroblast migration to the injury site. Activated fibroblasts undergo differentiation to myofibroblasts that synthesize collagen and other extracellular matrix proteins and play a pivotal role in wound healing [1]–[4]. However, unchecked wound healing leads to loss of tissue homeostasis due to excessive synthesis and deposition of collagens by activated myofibroblasts in the wound area, a pathological manifestation of organ fibrosis [1]–[6]. Fibrosis is an end-stage pathological manifestation of numerous injury related diseases where different deregulated biological processes such as inflammation, cellular migration, proliferation, senescence and cytokine signaling participate and contribute to disease progression [1], [2], [7], [8]. At present, there is no effective therapy for organ fibrosis. In order to develop a novel therapeutic approach, it is important to identify the molecule(s) which ignite(s) the onset of fibrogenesis. Previous studies demonstrated that aged PAI-1 knockout mice develop cardiac-specific fibrosis without affecting any other organ [8]–[10] and thus provide an excellent animal model for identification of factor(s) that ignite(s) organ-specific fibrosis.

Plasminogen activator inhibitor-1 (PAI-1), a member of the serine protease inhibitor (serpin) gene superfamily, is the major physiologic inhibitor of serine proteases, urokinase-type plasminogen activator (u-PA) and tissue-type plasminogen activator (t-PA). The PAI-1 gene, codes for 48–50 kDa polypeptides with two well-characterized functional domains: vitronectin binding domain required for anchoring PAI-1 to extracellular matrix and reactive center loop (RCL) required for inhibition of u-PA and t-PA activities. PAI-1 is synthesized by a wide variety of cells including endothelial cells, adipocytes, cardiomyocytes, fibroblasts and macrophages. Active PAI-1 controls several biological processes in a cell-type- and tissue-type-specific manner including cellular proliferation, migration, adhesion, apoptosis, senescence, blood coagulation via modulating the activities of cytokines and transcriptional regulators [5], [11]–[13]. Through inhibition of uPA/tPA, PAI-1 inhibits the plasminogen-to-plasmin conversion and plasmin-dependent matrix metalloproteinase (MMP) activation. Non-physiologic levels (both decreased and increased) of this secreted serine protease inhibitor are associated with a wide variety of human diseases such as obesity, insulin resistance, diabetes, cardiovascular diseases, abnormal bleeding, emphysema, thrombosis, atherosclerosis, cancer, impairment of wound healing and multi-organ fibrosis [5], [14], [15]. Numerous studies demonstrated that in injury-induced fibrotic tissues, the level of PAI-1 is significantly elevated and plays an important role in fibrogenesis via suppression of the proteolytic degradation of extracellular matrix proteins [5]. Furthermore, PAI-1 deficient mice are protected from bleomycin-induced lung fibrosis and unilateral ureteral obstruction (UUO)-induced renal fibrosis suggesting the positive role of PAI-1 in fibrogenesis [16], [17]. Paradoxically, PAI-1 deficiency is associated with age-dependent spontaneous cardiac selective fibrosis and is characterized by the presence of inflammation, elevated levels of TGF-ß, constitutively activated Smads and Erk1/2 MAPK, increased number of fibroblast like cells and excessive collagen accumulation in pericardial, perivascular and interstitial areas [8]–[10]. Furthermore, PAI-1-deficient endothelial cells are more susceptible to endothelial to mesenchymal transition (EndMT) and EndMT derived myofibroblast-like cells may contribute to elevated collagen synthesis and cardiac fibrogenesis (reviewed in [5], [8], [18]). However, the molecular basis of spontaneous cardiac-selective fibrosis in this murine model is an enigma.

As global PAI-1 knockout mice (the whole coding region and part of the promoter deleted) [19], [20] develop age-dependent spontaneous fibrosis only in the hearts (8–10), we asked the most fundamental questions: why are the kidney and other organs not affected by fibrogenesis in PAI-1 knockout mice? Why does only the PAI-1 knockout heart develop fibrosis? In other words, what are the factors that ignite fibrogenesis in the heart? What is the status of those factors in PAI-1 knockout kidneys? We hypothesized that specific regulator(s) involved in initiation of fibrogenesis are differentially expressed in the PAI-1 knockout heart and kidney. In order to address this prediction, we examined the global gene expression profile in hearts and kidneys derived from aged wildtype and PAI-1 knockout mice using Illumina-based microarray analysis. We have identified several genes that are differentially expressed in PAI-1 knockout hearts and kidneys compared to wildtype controls. Importantly, we have identified a number of important regulators related to matrix remodeling and cardiac hypertrophy, which are upregulated in PAI-1 knockout hearts compared to wildtype hearts and PAI-1 knockout kidneys. The significance of these novel findings in the light of cardiac-specific fibrogenesis is discussed.

## Materials and Methods

### Experimental Animals and Organ Collection

PAI-1 knockout (PAI-1KO) and wild-type (WT) mice on a C57BL/6 background were purchased from Jackson laboratory (Bar Harbor, ME) and were maintained for 12–24 months. All mouse protocols were approved by the Animal Care and Use Committee of Northwestern University (Chicago, IL). The mice were sacrificed at the age of 12–24 months and heart, kidney, liver and lung were collected. Part of the heart, kidney, liver and lung was fixed in formalin and stored for histochemical analysis. The other half of the organ was snap frozen and stored at −80°C for RNA and biochemical analysis.

### Histology and Collagen Staining

For histological studies, fixed hearts derived from wildtype and PAI-1 knockout mice (n = 3–5) were sectioned in the short axis along the mid ventricle. The fixed kidneys, livers and lungs were sectioned and processed for staining. The levels of collagen deposition in hearts and kidneys were determined by Masson’s trichrome staining. Photographs were taken with an Olympus DP71 camera as described [8]. For quantification of fibrotic areas, Masson’s trichrome stained sections (whole tissues or 5 fields/mice) were analyzed with Image-Pro-Plus 6.3 software (Media Cybernetics, Bethesda, MD).

### RNA Extraction from Hearts and Kidneys Derived from PAI-1 Knockout and Wildtype Mice

Total RNA was extracted from hearts and kidneys derived from three PAI-1 knockout (12- month old) and three wild-type mice (12-month old) using RNeasy Fibrous Tissue Mini Kit (Qiagen, Valencia, CA) following the manufacturer’s instructions. In short, heart and kidney tissues were homogenized in RLT buffer using Tissue Lyser. The tissue lysate was mixed with RNase-free water and proteinase K and incubated at 55°C for 10 min. The lysate was centrifuged and supernatant was mixed with ethanol and the mixture was loaded to RNeasy mini column and centrifuged. Buffer RW1 was added to the column and centrifuged. DNase in RDD buffer was added to the column and incubated for 15 min at 25°C. The RNeasy column was washed with RW1 buffer and RPE buffer. The total RNA was collected in a new centrifuge tube using 50 µl RNase-free water and centrifugation. The concentrations of total RNA in each heart tissue and kidney tissue were determined by spectrophotometric analysis. The quality of RNA (RNA Integrity, RIN) in all 12 samples (3 wildtype hearts; 3 PAI-1 KO hearts; 3 wildtype kidneys; and 3 PAI-1 KO kidneys) was checked using the bioanalyzer.

### First and Second cDNA Strand Synthesis: cRNA Synthesis

For first strand synthesis, T7 oligo(dT) Primer, 10X First Strand Buffer, dNTP Mix, and RNase inhibitor were added in a RNA containing tube (20 µl) and incubated at 42°C for 2 h. To prepare the second strand, nuclease free water, 10X Second Strand buffer, dNTP Mix, DNA polymerase, and RNaseH (80 µl) were mixed. The master mix was transferred to each first strand synthesized sample and incubated at 16°C for 2 h. **cDNA purification**: 250 µl of cDNA binding buffer was added to each sample and mixed thoroughly before placing onto the middle of the filter Cartridge. The sample was centrifuged for 2 min and the flow-through was discarded. The cDNA filter cartridge was washed with wash buffer. The sample was centrifuged and flow-through was discarded. The cartridge was transferred to the cDNA elution tube and 10 µl of warm (53°C) nuclease free water was added. The sample was incubated at room temperature for 2 min and was centrifuged to collect the cDNA. A second aliquot of 10 µl preheated nuclease free water was applied and centrifuged for 2 min. The tubes were kept on ice for further processing.

### *In vitro* Transcription Reaction and cRNA Purification

T7 10X Reaction Buffer, T7 Enzyme Mix, Biotin-NTP Mix (total volume 7.5 µl) were mixed and spun for a few seconds. 7.5 µl of the IVT mix was added to each purified cDNA sample and mixed well. The sample was incubated at 37°C for 14 h. 350 µl of cRNA binding buffer followed by 250 µl of ethanol was added to each sample and mixed. The mixture was transferred to the cRNA filter cartridge and centrifuged for 2 min. The flow-through was discarded and 650 µl wash buffer was added to each cRNA filter cartridge and centrifuged. The filter cartridge was transferred to the fresh cRNA collection tubes and 40 µl warm nuclease free water was added. The filter was incubated at room temperature for 2 min and centrifuged. The quality of the sample was checked and the concentration of the cRNA was measured using the bioanalyzer.

### Microarray Procedure: Illumina Gene Expression Analysis QC Report

Twelve samples were processed. All the samples met the Illumina standard quality control checks. Data quality checks were performed using Bioconductor Lumi package [21] for R statistical programming environment. The data processing also included a normalization procedure utilizing quantile normalization method [22] to reduce the obscuring variation between microarrays, which was introduced during the processes of sample preparation, manufacture, fluorescence labeling, hybridization and/or scanning. Hierarchical clustering and Principal Component Analysis were performed on the normalized signal data to assess the sample relationship and variability. Multiple metrics were taken to assess the overall quality of the hybridization. The advantage of quantile normalization is that it preserves the rank order of genes and has computation efficiency. However, it is possible that quantile normalization may eliminate differentially expressed genes whose intensity values are small.

### Relationship among and Variability of the Samples (Arrays)

**Figure 1:**
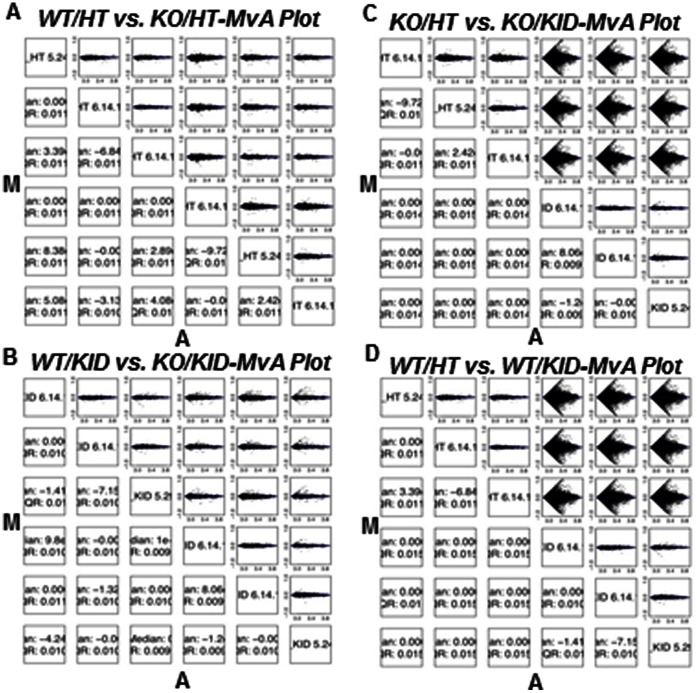
Relationship among and variability of samples. Hierarchical clustering and multi-dimensional scaling analyses were performed. The MvA plots showing the small variation in **A**. WT/HT vs. KO/HT and **B.** WT/KID vs. KO/KID, and large variation in **C.** KO/HT vs. KO/KID and **D.** WT/HT vs. WT/KID.

Hierarchical clustering and multi-dimensional scaling analyses were performed to assess the relationship and variability of the samples. All the 4 groups were clearly separated from each other. The MvA plots showed that there was a large variation between certain groups, such as Group2 (KO/HT) vs Group4 (KO/KID) and Group1 (WT/HT) vs. Group3 (WT/KID), suggesting big changes in gene expression. The biological replicates were very consistent. The variation between other groups, eg. Group1 (WT/HT) vs. Group2 (KO/HT) and Group3 (WT/KID) vs. Group4 (KO/KID) was very small (**See** **Figure 1A–D**). Based on the analyses above, the overall data quality for all the 12 samples was acceptable.

### Data Filtering and Microarray Gene Expression Analysis Method

As most genes are expressed only under certain conditions, lots of genes have expression signals below the background. These are defined by Illumina as “absent”. Probes absent in all samples were filtered out, leaving 19780 probes corresponding to 11105 genes in the downstream analysis. Differential gene expression between the different conditions was assessed by a statistical linear model analysis using the bioconductor package *limma,* in which an empirical Bayes method was used to moderate the standard errors of the estimated log-fold changes of gene expression. This method results in more stable inference and improved power, especially for experiments with small numbers of microarrays [23], [24]. The moderated t-statistic p-values derived from the *limma* analysis were further adjusted for multiple testing by Benjamini and Hochberg’s method to control false discovery rate (FDR). The lists of differentially expressed genes were obtained by the criteria of p-value <0.05 and fold change cutoff 1.5 for WT/HT vs KO/HT and WT/KID vs KO/KID and criteria of FDR <10% and fold change cutoff 1.5 for WT/HT vs WT/KID and KO/HT vs. KO/KID, and visualized by volcano plots. The microarray data has been deposited to NCBI GEO with the access number GSE46320.

### Quantitative qPCR of Genes for Validation: Procedure Real-Time Reverse-Transcription Polymerase Chain Reaction Analysis

The messenger RNA (mRNA) levels were quantified by complementary DNA synthesis (iScript cDNA Synthesis Kit, Bio-Rad, Hercules, CA) and quantitative polymerase chain reaction with SYBR Green SuperMix for IQ (Quanta Bioscience, Gaithersburg, MD). Levels of Ankrd1, Pi16, Egr1, Dbp and TIMP-1 were measured by quantitative PCR using gene specific primers and All-in-One qPCR kit (GeneCopoeia, Rockville, MD).

### Endothelial to Mesenchymal Transition (EndMT) Study and Western Blot Analysis

Mouse cardiac endothelial cells were cultured in 10% FBS containing EBM-2 media. The cells were cultured overnight at 2% FBS containing media and treated with TGF-ß2 for 7days. At the end of the incubation, cells were harvested and whole cell lysates were prepared. Equal amount of proteins were loaded on a 4–12% Tris-glycine gradient gel and processes for immunoblot analysis as described [8] using antibodies (1 µg/ml) against Ankrd1 (Novus Biologicals, Littleton, CO), Pi16 (R&D, Minneapolis, MN), Timp1 (Santa Cruz Biotech, Santa Cruz, CA), Timp4 (Millipore, Billerica, MA) and Actin (Abcam, Cambridge, MA).

### Statistical Analysis

Data are presented as Mean ± SEM. The significance of differences between controls and experimental groups were estimated by t-test and a value of *P*<0.05 by Student *t* test was considered statistically significant. Statistical analyses were performed with GraphPad Prism 3.0 (GraphPad Software Inc, San Diego, CA).

## Results

### Pathological Manifestation of Fibrosis in Hearts and Kidneys Derived from 12-month and 24-month Old Wildtype and PAI-1 Knockout Mice

**Figure 2:**
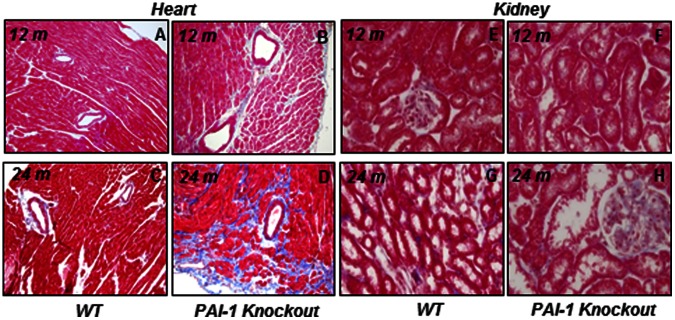
PAI-1 deficiency is associated with fibrosis in hearts but not in kidneys. The levels of collagen accumulation in cardiac and renal tissues derived from wildtype and PAI-1 knockout mice were determined by Masson’s trichrome staining. Photographs were taken by an Olympus DP71 camera. **A.** 12-month old WT/HT; **B.** 12-month old PAI-1 KO/HT; **C.** 24-month old WT/HT; **D.** 24-month old PAI-1 KO/HT; **E.** 12-month old WT/KID, **F.** 12-month old PAI-1 KO/KID, **G.** 24-month old WT/KID, **H.** 24-month old PAI-1 KO/KID.

Previously, we reported that PAI-1 deficiency is associated with age-dependent cardiac fibrosis [8]. Hearts and kidneys explanted from aged PAI-1 knockout and wildtype mice were subjected to histological study. Levels of collagen were measured by Masson’s trichrome staining. Results revealed that while myocardial tissues derived from 12-month old PAI-1 knockout mice showed modest accumulation of collagen compared to age-matched wildtype controls (Wildtype 12 m 0.0057±0.0015 vs. PAI-1 knockout 12 m 0.0541±0.0248; p = 0.09), myocardial tissues derived from 24-month old PAI-1 knockout mice showed significantly elevated levels of collagen accumulation compared to age- and sex-matched wildtype controls (Wildtype 24 m 0.0143±0.006 vs. PAI-1 knockout 24 m 0.1546±0.0144; p = 0.0004) (**Figure 2A–D**). In contrast, collagen accumulation in kidneys derived from both 12-month and 24-month old PAI-1 knockout mice were insignificant and comparable with age- and sex-matched wildtype controls (Wildtype 12 m 0.00125±0.00029 vs. PAI-1 knockout 12 m 0.00153±0.00035; p = 0.55) (Wildtype 24 m 0.00248±0.00052 vs. PAI-1 knockout 24 m 0.00177±0.00033; p = 0.24) (**Figure 2E–H**) suggesting PAI-1 deficiency is associated with age-dependent cardiac-selective fibrosis. Results also revealed that PAI-1 deficiency is not associated with age-dependent fibrosis in liver (Wildtype 24 m 0.00203±0.00051 vs. PAI-1 knockout 24 m 0.00125±0.00017; p = 0.11) and lung (Wildtype 24 m 0.00209±0.00054 vs. PAI-1 knockout 24 m 0.00196±0.00054; p = 0.86) compared to age-matched wildtype controls (**Figure S1**). As the molecular events of fibrogenesis start earlier, before the pathological manifestation of fibrosis, we decided to study the global gene expression profiling in 12-month old PAI-1 knockout mice where pathological manifestation of fibrosis or accumulation of collagen is not significant. We anticipated that this would help us to identify the regulatory factors responsible for initiation of fibrogenesis in the heart.

### Hierarchical Clustering of Gene Expression: Summary and Interpretation of the Comparisons with Statistics

**Figure 3:**
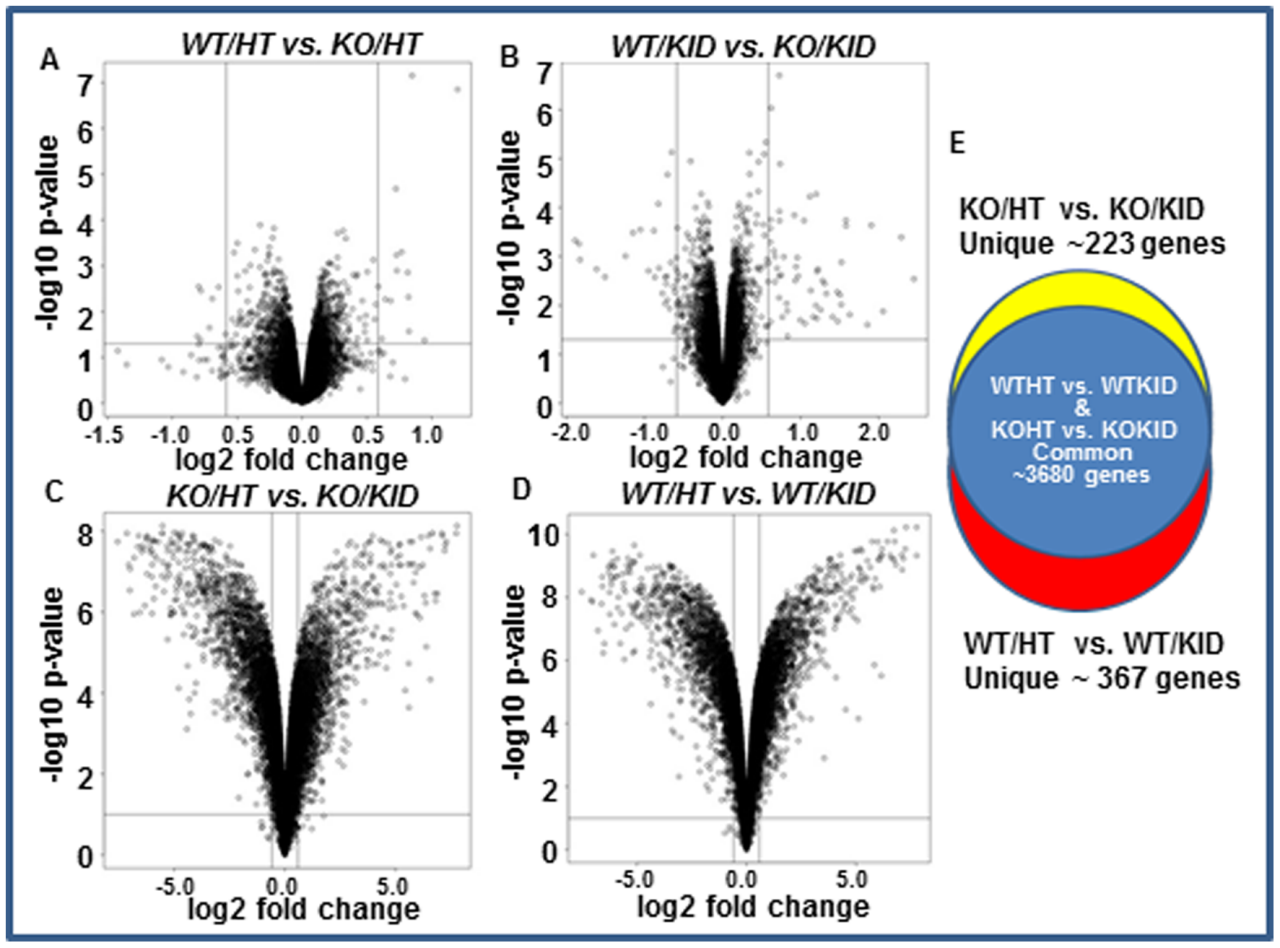
Effect of PAI-1 deficiency on differential global gene expression profiling in hearts and kidneys. Differentially expressed genes are presented by volcano plots with fold induction or repression and statistical significance in four different groups. **A.** Expression levels of genes in wildtype and PAI-1 knockout hearts. **B.** Expression levels of genes in wildtype and PAI-1 knockout kidneys. **C.** Expression levels of genes in PAI-1 knockout hearts and kidneys. **D.** Expression levels of genes in wildtype hearts and kidneys. **E.** Venn diagram showing the differentially expressed genes which are common in PAI-1 KO/HT vs. PAI-1 KO/KID group and WT/HT vs. WT/KID group (Blue); and unique in PAI-1 KO/HT vs. KO/KID group (Yellow) and WT/HT vs. WT/KID group (Red).

The global gene expression profile in wildtype and PAI-I knockout hearts and kidneys were compared. Four comparisons were made within four groups: Wildtype heart (WT/HT), Knockout Heart (KO/HT), Wildtype Kidney (WT/KID) and Knockout Kidney (KO/KID). We compared Group1 (WT/HT) with Group2 (KO/HT), Group3 (WT/KID) with Group4 (KO/KID), Group2 (KO/HT) with Group4 (KO/KID) and Group1 (WT/HT) with Group3 (WT/KID) respectively (**Figure 3**). For the comparison, the first condition in the group name before the “vs.” sign is upregulated when the fold change has a positive, and is down-regulated when the fold change has a negative sign**.** The results of the probe-level differential gene expression were visualized by a volcano plot. While only a few genes were found to be differentially expressed between WT/HT and KO/HT (Figure 3A) and between WT/KID and KO/KID (Figure 3B), the expression patterns of numerous genes were significantly different when compared between KO/HT and KO/KID (**Figure 3C**) or between WT/HT and WT/KID (Figure 3D) as visualized by volcano plots.

### Differentially Expressed Genes

Global gene expression profiling revealed that only 17 genes were differentially expressed in wildtype and PAI-1 knockout hearts. Out of 17 genes, 7 genes including Ankrd1, Dbp, Fbln2, Pi16 and Phlda 1 were upregulated and 10 genes including Acta2, H2-Ab1, Spon2 and Ces1d were downregulated in PAI-1 knockout hearts compared to age- and sex-matched wildtype controls (**Table 1** **;** see **Table S1**). Gene expression profiling revealed that 31 genes were differentially expressed in kidneys derived from PAI-1 knockout mice and wildtype mice. Out of 31 differentially expressed genes, 7 genes were upregulated including Prlr, Acad10, Aldh8a1 and 24 genes were downregulated including ler3, Egr1, Bcl6 and Dbp in PAI-1 knockout kidneys compared to age- and sex-matched control kidneys (**Table 2** **;** for details see **Table S2**). Almost 3900 genes were differentially expressed when compared between knockout hearts and knockout kidneys (**Table S3**). Many of these differentially expressed genes in knockout hearts and kidneys represent tissue-specific differential gene expression as well as differential expression influenced by PAI-1 deficiency as evidenced by altered fold change of expression of a particular gene when compared between WT/HT vs. WT/KID with KO/HT vs. KO/KID. While gene expression profiling was compared between wildtype hearts and kidneys, 4049 genes were differentially expressed (**Table S4**). These differentially expressed genes in wildtype hearts and kidneys represent only tissue-specific differential gene expression. This is one of the limitations of comparing of global gene expression profiling using two different tissues. However to overcome this issue, we further identified unique genes which are differentially expressed either only in the knockout heart and kidney comparison or in the wildtype heart and kidney comparison. Results revealed that while the expression of 223 genes out of 3900 differentially expressed common genes were altered uniquely in knockout heart and kidney (**Table 3** **, Table S5,** **Figure 3E**), 367 genes out of 4049 differentially expressed common genes showed altered expression uniquely in wildtype heart and kidney. For example, Agtr 1a, Vegfc, Sirt2, Rb1 were upregulated and Ctgf, Ier3, Junb were downregulated in wildtype heart compared to wildtype kidney (**Table 4** **, Table S6;** **Figure 3E**). Please note that the genes listed in **Table 3** and **Table 4** were unique in two comparisons. As the primary goal of the present study was to identify the differentially expressed genes related to matrix remodeling, we further analyzed the results of unique gene expression profiling in knockout hearts and kidneys (absent in wildtype heart and kidney comparison). We have identified several upregulated genes including Timp1, Dbp, Scx, MMP23, Egr1, Ebf2, Klf6 and Loxl1 (**Table 3A**) in knockout hearts compared to knockout kidneys which may individually or together play an important role in matrix remodeling and cardiac fibrosis. The downregulated genes in knockout hearts compared to knockout kidneys include Xbp1, Stx7, Dad1, Cdk8 and Map3k11 (**Table 3B**).

**Table 1:** Differential gene expression in wildtype and PAI-1 knockout hearts.

Gene Symbol	Gene Name Upregulated (A)	p value
Ankrd1	Ankyrin repeat domain1	0.04
Dbp	D site albumin binding protein	0.03
Fxyd6	FXYD domain containing ion transport regulator 6	0.003
Fbln2	Fibulin 2	0.03
Pi16	Protease inhibitor 16	0.02
Phlda 1	Pleckstrin homology-like domain family Amember 1	0.002
**Gene Symbol**	**Gene Name Downregulated (B)**	**p value**
Taf6	Transcription initiation factor TFIID subunit 6	0.00002
H2-Ab1	Histocompatibility 2, class II antigen A, beta 1	0.03
Zfp68	Zinc finger protein 68	0.0000001
Acot 1	Acyl-CoA thioesterase 1	0.02
Spon2	Spondin 2	0.001
Ces1d	Carboxylesterase 1D	0.04

Genes upregulated (>1.5 fold) in knockout hearts compared to wildtype hearts (A). Genes downregulated (<1.5 fold) in knockout hearts compared to wildtype hearts (B).

**Table 2:** Differential gene expression in wildtype and PAI-1 knockout kidneys.

GeneSymbol		Gene Name Upregulated (A)	p value
Aldh8a1		Aldehyde dehydrogenase 8 family, member A1	0.02
Prlr		Prolactin receptor	0.00002
Hbb-b1		hemoglobin beta, adult major chain	0.001
Hbb-a1		hemoglobin alpha, adult chain 1	0.03
Acad10		Acyl-coenzyme dehydrogenase family, member 10	0.001
Dnase 1		deoxyribonuclease 1	0.009
**Gene** **Symbol**	**Gene Name Downregulated (B)**		**p value**
Dusp1	Dual specificity phosphatase 1		0.002
Ier3	Immediate early response 3		0.003
Zfp36	Zinc finger protein 36		0.001
Zfp68	Zinc finger protein 68		0.000002
JunB	JunB oncogene		0.007
Prf1	perforin/serine protease		0.04
Egr1	Early growth response 1		0.0001
Bcl6	B cell lymphoma 6		0.02
Dbp	D-site albumin promoter binding protein		0.02
Mvp	major vault protein		0.005
Chka	Choline kinase alpha		0.009

Genes upregulated (>1.5 fold) in knockout kidneys compared to wildtype kidneys (A). Genes downregulated (<1.5 fold) in knockout kidneys compared to wildtype kidneys (B).

**Table 3:** Differential gene expression in PAI-1 knockout hearts and kidneys.

GeneSymbol	Gene Name Upregulated (A)	p value
TIMP1	Tissue inhibitor of metalloproteinase 1	0.02
Dbp	D site albumin promoter binding protein	0.01
Scx	Scleraxis	0.001
Col3A1	Collagen, type III, alpha 1	0.007
MMP23	Matrix metallopeptidase 23	0.01
Igfbp6	Insulin-like growth factor binding protein 6	0.005
Klf6	Kruppel-like factor 6	0.00005
Notch4	Notch gene homolog 4	0.0003
Ebf2	Early B cell factor 2	0.00005
Timp2	Tissue inhibitor of metalloproteinase 2	0.008
Egr1	Early growth response 1	0.0002
Dusp1	Dual specificity phosphatase 1	0.00001
Loxl1	Lysyl oxidase-like 1	0.02
il6st	Interleukin 6 signal transducer	0.0002
**Gene** **Symbol**	**Gene Name Downregulated (B)**	**p value**
Xbp1	X-box binding protein 1	0.0005
Stx7	Syntaxin 7	0.003
Map3k11	Mitogen activated protein kinase kinase kinase 11	0.0001
Zfp523	Zinc finger protein 523	0.00001
Dad1	Defender against cell death 1	0.0001
Cdk8	Cyclin-dependent kinase 8	0.00004

Genes upregulated (>1.5 fold) in knockout hearts compared to knockout kidneys (unique) (A). Genes downregulated (<1.5 fold) in knockout hearts compared to knockout kidneys (unique) (B).

**Table 4:** Differential gene expression in wildtype hearts and kidneys (Unique).

GeneSymbol	Gene Name Upregulated (A)	p-value
Agtr1a	angiotensin II receptor, type 1a	0.000002
Vegfc	vascular endothelial growth factor C	0.0000008
Hbb-b1	hemoglobin, beta adult major chain	0.001
Taf9	TAF9 RNA polymerase II, TBP associated factor 9	0.000002
Fgf1	fibroblast growth factor 1	0.0004
Foxn3	forkhead box n3	0.000001
Rb1	retinoblastoma 1	0.00001
Tbrg1	transforming growth factor beta regulated gene 1	0.00002
Sirt2	sirtuin 2	0.00002
Apoe	apolipoprotein E	0.03
Mdm2	transformed mouse 3T3 cell double minute	0.0000002
nfkb1	nuclear factor of kappa light polypeptide	0.0000001
Ier3ip1	immediate early response 3 interacting protein1	0.0000005
Prlr	prolactin receptor	0.000002
Nfatc1	nuclear factor of activated T-cells	0.00003
**Gene** **Symbol**	**Gene Name Downregulated (B)**	**p-value**
Zfp36	zinc finger protein 36	0.0004
Ctgf	connective tissue growth factor	0.0006
Dusp6	dual specificity phosphatase 6	0.0001
Ubtf	upstream binding transcription factor	0.00002
Ier3	immediate early response 3	0.00001
Dapk2	death-associated protein kinase 2	0.000003
Sdc2	syndecan 2	0.000005
Mapk13	mitogen-activated protein kinase 13	0.0000004
Junb	Jun-B oncogene	0.03

Genes upregulated (>1.5 fold) in wildtype hearts compared to wildtype kidneys (A). Genes downregulated (<1.5 fold) in wildtype hearts compared to wildtype kidneys (B).

### Biological Processes Representing Genes that were Differentially Expressed in Wildtype and PAI-1 Knockout Hearts

**Figure 4:**
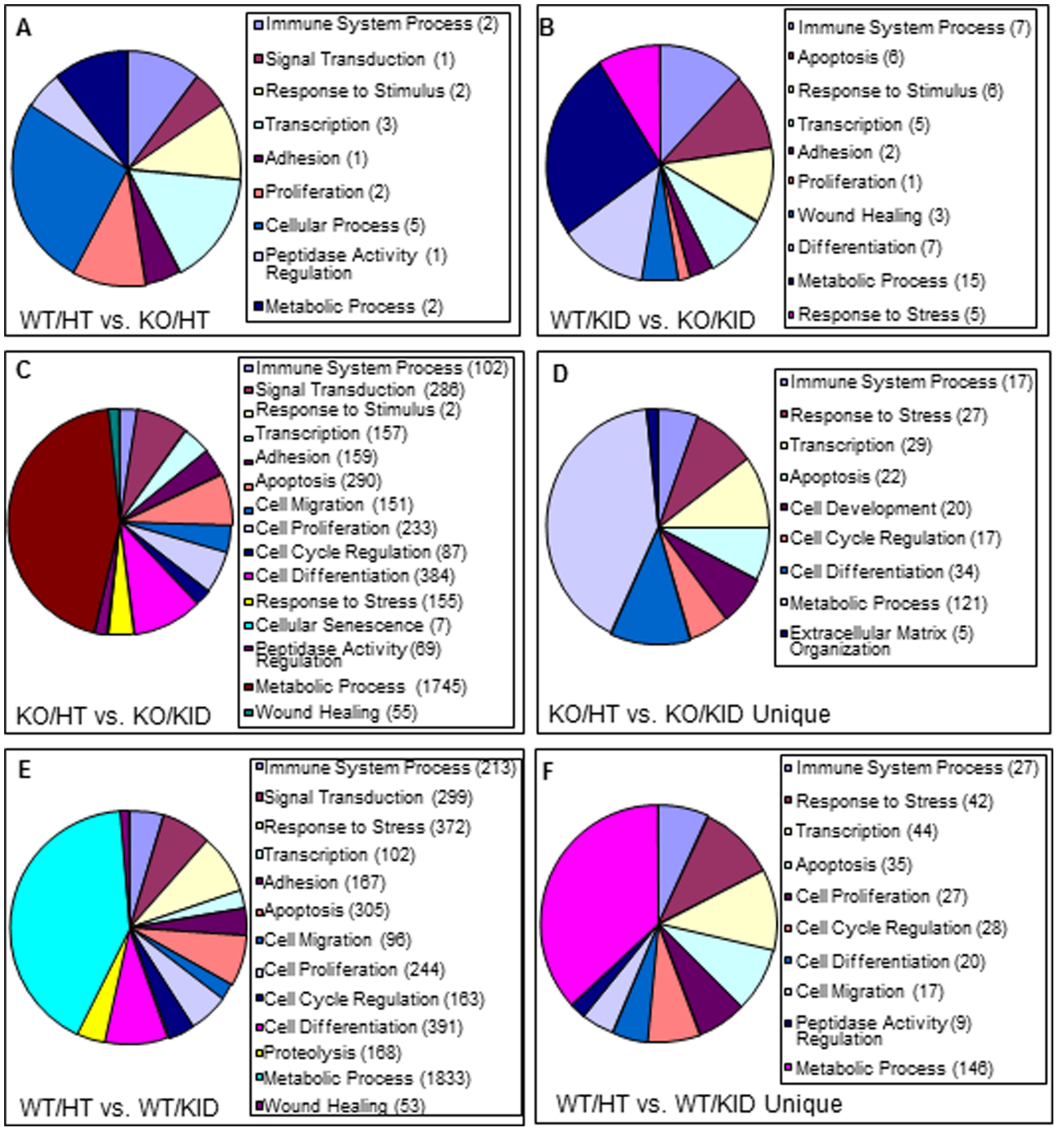
Biological processes representing genes that were differentially expressed in wildtype and PAI-1 knockout heart and kidney groups. Percentages of differentially expressed genes (upregulated or downregulated) under different biological processes are shown by Pie charts. **A.** WT/HT vs. PAI-1 KO/HT; **B.** WT/KID vs. PAI-1 KO/KID; **C.** PAI-1 KO/HT vs. PAI-1 KO/KID and **D.** PAI-1 KO/HT vs. PAI-1 KO/KID (unique); **E.** WT/HT vs. WT/KID; **F.** WT/HT vs. WT/KID (unique). Numbers of deregulated genes are shown in parentheses.

Genes involved in a wide variety of biological processes were deregulated in aged PAI-1 knockout hearts compared to age- and sex- matched wildtype controls. The biological processes depicting genes that were deregulated by the absence of PAI-1 are shown in **Table 5** and **Figure 4A**. For examples: Ankrd1 which is involved in signal transduction, transcriptional regulation, and wound healing biological processes was upregulated in PAI-1 knockout hearts compared to wildtype hearts. Pi16 gene which is involved in the regulation of peptidase activity biological process and cardiac hypertrophy was also upregulated in PAI-1 knockout hearts compared to wildtype controls. H2-Ab1 and Spon2 genes, which are involved in immune response, were deregulated.

**Table 5:** Biological Processes representing genes that were differentially expressed in knockout hearts compared to wildtype hearts (for level of expression of each gene see Table S1).

Gene Ontology ID	Biological Processes	Gene Symbols
**GO:0045087**	**innate immune response**	H2-Ab1, Spon2
**GO:0030330**	**DNA damage response, signal transduction…**	Ankrd1
**GO:0034097**	**response to cytokine stimulus**	H2-Ab1, Ankrd1
**GO:0006366**	**transcription from RNA polymerase II promoter**	Taf6, Ankrd1, Dbp
**GO:0010811**	**positive regulation of cell-substrate ad…**	Fbln2
**GO:0008285**	**negative regulation of cell proliferation**	Taf6, H2-Ab1
**GO:0048522**	**positive regulation of cellular processes**	H2-Ab1, Ankrd1, Dbp, Fbln2, Phlda1
**GO:0052547**	**regulation of peptidase activity**	Pi16
**GO:0006732**	**coenzyme metabolic process**	Acot1, Ces1d

### Biological Processes Representing Genes that were Differentially Expressed in Wildtype and PAI-1 Knockout Kidneys

Genes involved in different biological processes were also deregulated in PAI-1 knockout kidneys compared to age- and sex-matched wildtype controls. The biological processes depicting genes that were deregulated in kidneys due to PAI-1 deficiency are shown in **Table 6** and **Figure 4B** **.** For examples: The genes Hbb-b1, Hba-a1, JunB, Egr1 and Bcl6 involved in immune system process and matrix remodeling were deregulated. The expression levels of genes involved in cell death, cell adhesion, proliferation, response to stress and wound healing biological processes were altered in the absence of PAI-1 suggesting these biological processes in kidneys are controlled directly or indirectly by PAI-1.

**Table 6:** Biological Processes representing genes that were differentially expressed in knockout kidneys compared to wildtype kidneys (for level of expression of each gene see Table S2).

Gene ontology ID	Biological Processes	Gene Symbols
**GO:0002376**	**immune system process**	Hbb-b1, Hba-a1, Zfp36, Junb, Prf1, Egr1, Bcl6
**GO:0008219**	**cell death**	Dusp1, Ier3, Prf1, Dnase1, Egr1, Bcl6
**GO:0030154**	**cell differentiation**	Prlr, Hbb-b1, Hba-a1, Zfp36, Junb, Egr1,Bcl6
**GO:0030155**	**regulation of cell adhesion**	Prlr, Bcl6
**GO:0030890**	**positive regulation of B cell proliferation**	Bcl6
**GO:0006950**	**response to stress**	Hbb-b1, Ier3, Zfp36, Prf1, Bcl6
**GO:0048583**	**regulation of response to stimulus**	Dusp1, Hbb-b1, Ier3, Zfp36, Egr1, Bcl6
**GO:0009611**	**response to wounding**	Ier3, Zfp36, Bcl6
**GO:0006366**	**transcription from RNA pol II promoter**	Zfp36, Junb, Egr1, Bcl6, Dbp
**GO:0043487**	**regulation of RNA stability**	Zfp36
**GO:0044238**	**primary metabolic process**	Aldha1, Dusp1, Rhbdl2, Rpl23, Prlr, Hbb-b1, Ier3, Zfp36, Hsd3b2, Junb, Dnase1, Egr1, Bcl6, Chka, Dbp

### Biological Processes Representing Genes that were Differentially Expressed in PAI-1 Knockout Hearts and Kidneys

Numerous genes involved in a variety of biological processes were deregulated in aged PAI-1 knockout murine hearts when compared to the gene expression profile of PAI-1 knockout murine kidneys. Numerous genes involved in metabolism, cell proliferation, cell migration, cell adhesion, cell cycle, apoptosis, response to stress and wound healing were deregulated (**Figure 4C** and data not shown). We further analyzed the biological processes for the genes which were uniquely expressed only in the KO/HT vs. KO/KID comparison and not in the WT/HT vs. WT/KID comparison. The biological processes depicting genes (unique) that were deregulated by the absence of PAI-1 are shown in **Table 7** and **Figure 4D**. Many of these deregulated genes are involved in transcriptional regulation, cellular differentiation and matrix organization including TIMP1, Scx, COL3A1, MMP23, IGFBP6, Klf6, Notch4, Timp2, Egr1, Dbp, Loxl1 and Il6st.

**Table 7:** Biological Processes representing genes that were differentially expressed in knockout hearts compared to knockout kidneys (unique) (for level of expression of each gene see Table S5).

Gene ontology ID	Biological Processes	Gene Symbols
**GO:0008152**	**metabolic process**	Timp1, Nudt18, Herc4, Ddx17, Dbp, Plk2, Elovl6, Scx, Gstm1, Tlk2, Xbp1, Ube2q1, Mbl1, Hnrpdl, Gstm5, Srd5a3, Cnksr1, Zranb1, Rgs10, Rgnef, Mrpl14, Kdm4b, Car3, Mrap, Cant1, Col3a1, Mrpl28, Mmp23, Zadh2, Map3k11, Aifm2, Bcl2l1, Irx2, Ddx39b, Adck1, Hmgn1, Ptpn21, Asnsd1, Mrps17, Irx5, Tgfbr1, Ebf2, Polr1e, Ndufv1, Dab2ip, Pja1, Unc45a, Etf1, Pigx, Timp2, Ei24, Fth1, Egr1, Zfp238, Nudt1, Hus1, Uqcr11, Dusp1, Nhp2, Ddit3, Guk1, Nr1d2, Klf6, Notch4, Timeless, Gns, Stk32a, Acot3, Scp2, Zfp523, Dad1, Hs3st1, Hras1, Csrnp1, Rnmtl1, Atxn1l, Rhox5, Rprd1a, Gpt2, Pdp2, Sqrdl, Ndufa1, Cdk8, Flad1, Il6st, Mme, Acd, Hdgfrp2, Htra1, Ggct, Avpr1a, Prkag1, Rcan1, Tmem68, Acvr2b, Rbp1, Bcat2, Chn2, Hmgcs2, Pfdn2, Anapc4, Nt5c3, Nr1d1, Uhrf2, Fut8, Map2k3, Fuk, Mertk, Ppip5k1, C4b, Aldh18a1, Fahd1, Uba1, Rnf19a, Akap6, Rsad1, Picalm, Alas1, Gpx6, Loxl1, Bag2
**GO:0007049**	**cell cycle**	Plk2, Tlk2, Map3k11, Bcl2l1, Timp2, Hus1, Dusp1, Ddit3, Nudc, Timeless, Hras1, Cdk8, Tacc2, Mad2l1bp, Anapc4, Pcbp4, Uhrf2
**GO:0008219**	**cell death**	Timp1, Plk2, Scx, Ypel3, Map3k11, Aifm2, Bcl2l1, Tgfbr1, Dab2ip, Ei24, Fth1, Egr1, Dusp1, Ddit3, Dad1, Hras1, Csrnp1, Rhox5, Itm2b, Mcl1, Pcbp4, Slc40a1
**GO:0030154**	**cell differentiation**	Timp1, Herc4, Ddx17, Scx, Tlk2, Xbp1, Rgnef, Mrap, Bcl2l1, Irx5, Shroom3, Tgfbr1, Dab2ip, Grn, Unc45a, Shank3, St7l, Timp2, Egr1, Notch4, Pldn, Rhox5, Lama2, Il6st, Tacc2, Pvrl2, Rcan1, Acvr2b, Rbp1, Mcl1, Snta1, Uhrf2, Mertk, Picalm
**GO:0006950**	**response to stress**	Tlk2, Xbp1, Mbl1, Car3, Aqp4, Col3a1, Map3k11, Hmgn1, Dab2ip, Shank3, Fgg, Nudt1, Hus1, Ddit3, Timeless, Pldn, Hras1, Stab1, Acd, Pvrl2, Avpr1a, Prkag1, Slc12a2, Pcbp4, Map2k3, Mertk, C4b
**GO:0048468**	**cell development**	Timp1, Xbp1, Rgnef, Bcl2l1, Irx5, Shroom3, Tgfbr1, Dab2ip, Grn, Shank3, Timp2, Notch4, Rhox5, Lama2, Il6st, Pvrl2, Rcan1, Snta1, Mertk, Picalm
**GO:0030198**	**extracellular matrix organization**	Scx, Crispld2, Col3a1, Tgfbr1, St7l
**GO:0006355**	**regulation of transcription, DNA-dependent**	Ddx17, Dbp, Scx, Xbp1, Hnrpdl, Kdm4b, Irx2, Irx5, Tgfbr1, Ebf2, Dab2ip, Egr1, Zfp238, Ddit3, Nr1d2, Klf6, Notch4, Timeless, Zfp523, Hras1, Csrnp1, Atxn1l, Rhox5, Rprd1a, Cdk8, Acvr2b, Nr1d1, Map2k3, Picalm
**GO:0002376**	**immune system process**	Timp1, Mbl1, Aqp4, Col3a1, Podxl2, Tgfbr1, Egr1, H2-Eb1, Pldn, Il6st, Pvrl2, Rbp1, Nr1d1, Mertk, C4b, Picalm, Slc40a1
**GO:0045446**	**endothelial cell differentiation**	Tgfbr1, Notch4
**GO:0016049**	**cell growth**	Tgfbr1, Dab2ip, St7l, Ei24, Htra1, Avpr1a

### Biological Processes Representing Genes that were Differentially Expressed in Wildtype Hearts and Kidneys

The biological processes representing numerous genes were also differentially expressed (upregulated and downregulated) in wildtype heart and kidney. This result was expected as numerous genes involved in metabolism, stress response and proliferation biological processes were differentially expressed in hearts and kidneys simply due to cell-type and tissue-specific expression (**Figure 4E** and data not shown). We further analyzed the biological processes for the genes which are uniquely expressed only in the WT/HT vs. WT/KID comparison but not in the KO/HT vs. KO/KID comparison are shown in **Table 8** and **Figure 4F**. Results showed that differentially expressed genes are involved in several biological processes including metabolic process, apoptosis, cell cycle, proliferation, migration, differentiation, immune system processes and response to stress. However, the majority of the differentially expressed genes in the WT/HT vs. WT/KID comparison represent tissue specific metabolic processes.

**Table 8:** Biological Processes representing genes that were differentially expressed in wildtype hearts compared to wildtype kidneys (unique) (for level of expression of each gene see Table S6).

Gene ontology ID	Biological Processes	Gene Symbols
**GO:0008152**	**metabolic process**	Mrpl51, Ctsc, Tcp1, Herpud1, Parp14, Prlr, Sgpl1, Mgrn1, Tpm2, Chka, Rps26, Amy1, Ahcyl1, Echs1, Zfp36, Mrpl36, Lypla1, Rnf10, Sgsm2, Rps2, Akap1, Kcmf1, Aip, Rangap1, Sars, Vegfc, Parp2, Akr1b3, Ehd1, Hdhd3, Eif2s3y, Fhit, Hbb-b1, Dnajb4, Tnfaip1, Pck1, Taf9, Pcdh12, Srrt, Ssbp2, Rpl3, Tfrc, Pold2, Trim8, Mdm2, Gucy1a3, Cul3, Galntl4, Polr1c, Pde4a, Ucp3, Exosc5, Spcs1, Atg16l1, Ctgf, Wwp2, Mrpl13, Serinc1, Apoe, Paox, Zfp187, Tef, Rhbdl2, Endod1, Atp9b, Cdk10, Pole3, Gngt2, Ndufc2, Pes1, Prkx, Dus4l, Wdyhv1, Fas, Eif4enif1, Spag9, Abcb6, Tbx10, Adck4, Rgs4, Ddx3y, Rpl23, Meis2, Il34, Mrpl22, Mcf2l, Eci2, Rars2, Kif20b, Diablo, C1rl, Haghl, Usp18, Hat1, Med4, Cln6, Ccnd1, Rgl2, Zfp691, Fbp2, Phkb, Brms1, Cpt2, Ext1, Rpl18a, Stk30, Rps6ka1, Gab1, Notum, Mrpl20, Nfkb1, H2-M3, Fgf1, Foxn3, Flcn, Rpl27a, Gpx4, Ranbp1, Tnni2, Nfatc1, Dusp6, Abp1, Zdhhc9, Bap1, Bnip1, Hint2, Eif3d, Npm1, Rb1, Agtr1a, Sphk1, Vps35, Zfp770, Sep15, Caprin1, Cyth1, Dnajc24, March5, Csdc2, Tbrg1, Mgst1, Ppip5k2, Mark3, Zfp771, Ier3, Gstk1
**GO:0008219**	**cell death**	Ctsc, Herpud1, Ltbr, Sgpl1, Taf9, Mdm2, Maea, Ctgf, Apoe, Zfp346, Fas, Diablo, Brms1, Rps6ka1, Nfkb1, Dusp6, Ier3ip1, Bnip1, Hint2, Rb1, Sphk1, Dram2, Ier3, Ptk2b, Eif5a, Katnb1, Prf1, Rhot1, Hspd1, Casp9, Bad, Tsta3, Insl6, Mmd, Dapk2
**GO:0007049**	**cell cycle**	Calm2, Mdm2, Cul3, Maea, Ctgf, Cdk10, Pes1, Appl2, Kif20b, Ccnd1, Stk30, Foxn3, Ranbp1, Nfatc1, Bap1, Npm1, Rb1, Sphk1, Tbrg1, Katnb1, Junb, Mapk13, Bad, Psmd13, Rhoc, Insl6, Sirt2, Nipbl
**GO:0006950**	**response to stress**	Herpud1, Ltbr, Vapb, Zfp36, Parp2, Akr1b3, Hbb-b1, Mdm2, Ucp3, Pvr, Atg16l1, Ctgf, Apoe, Fas, Spag9, Il34, C1rl, Ccnd1, Gab1, Nfkb1, H2-M3, Fgf1, Gpx4, Abp1, Mustn1, Npm1, Agtr1a, Sphk1, Ier3, Ptk2b, Prf1, Alas2, Fosl2, Hspd1, Mapk13, Pdia5, Cxcl9, Casp9, Mid1, Hmox2, Irgm2, Nipbl
**GO:0002376**	**immune system** **process**	Ctsc, Ltbr, Sgpl1, Zfp36, Vegfc, Hbb-b1, Tfrc, Pvr, Maea, Prkx, Fas, Il34, C1rl, Nfkb1, H2-M3, Rb1, Hba-a1, Ptk2b, Prf1, Alas2, Junb, Hspd1, Cxcl9, Bad, Ptpre, Bcar1, Irgm2
**GO:0008283**	**cell proliferation**	Rnf10, Vegfc, Akr1b3, Srrt, Ctgf, Apoe, Pes1, Prkx, Fas, Appl2, Il34, Tmem127, Ccnd1, H2-M3, Fgf1, Bap1, Npm1, Rb1, Sphk1, Tbrg1, Ptk2b, Eif5a, Junb, Fosl2, Hspd1, Akr1c21, Bad
**GO:0016477**	**cell migration**	Cap1, Sgpl1, Vegfc, Tnfaip1, Cul3, Pvr, Ctgf, Apoe, Prkx, Spag9, Gab1, Fgf1, Agtr1a, Sphk1, Ptk2b, Bcar1, Cyp1b1
**GO:0006355**	**regulation of** **transcription,** **DNA-dependent**	Parp14, Zfp36, Rnf10, Taf9, Ssbp2, Mdm2, Wwp2, Zfp187, Tef, Tbx10, Meis2, Mcf2l, Hat1, Med4, Zfp691, Brms1, Rps6ka1, Nfkb1, Fgf1, Foxn3, Tnni2, Nfatc1, Npm1, Rb1, Sphk1, Zfp770,Csdc2, Zfp771, Ubtf, Junb, Mterfd1, Zfp27, Arid3a, Fosl2, Mapk13, Smarce1, Ascc2, Thap11, Srebf2, Cbx3, Atf7ip, Dkk3, Ppp2r5d, Sirt2, Nipbl
**GO:0045595**	**regulation of cell** **differentiation**	Prlr, Zfp36, Rnf10, Vegfc, Ctgf, Prkx, Fas, Spag9, Ccnd1, H2-M3, Dusp6, Rb1, Sphk1, Caprin1, Ptk2b, Eif5a, Katnb1, Junb, Bad, Ppp2r5d
**GO:0052547**	**regulation of** **peptidase activity**	Herpud1, Mdm2, Ctgf, Diablo, Rps6ka1, Hspd1, Casp9, Bad, Itih3
**GO:0048468**	**cell development**	Rnf10, Vegfc, Hbb-b1, Gm16517, Cul3, Maea, Whrn, Fas, Spag9, Ext1, Nfatc1, Naglu, Rb1, Sphk1, Sep15, Caprin1, Hba-a1, Ptk2b, Katnb1, Agpat6, Bad, Ppp2r5d, Myh11, Insl6, Kcnip2

### Validation of Five Differentially Expressed Genes by qPCR

**Figure 5:**
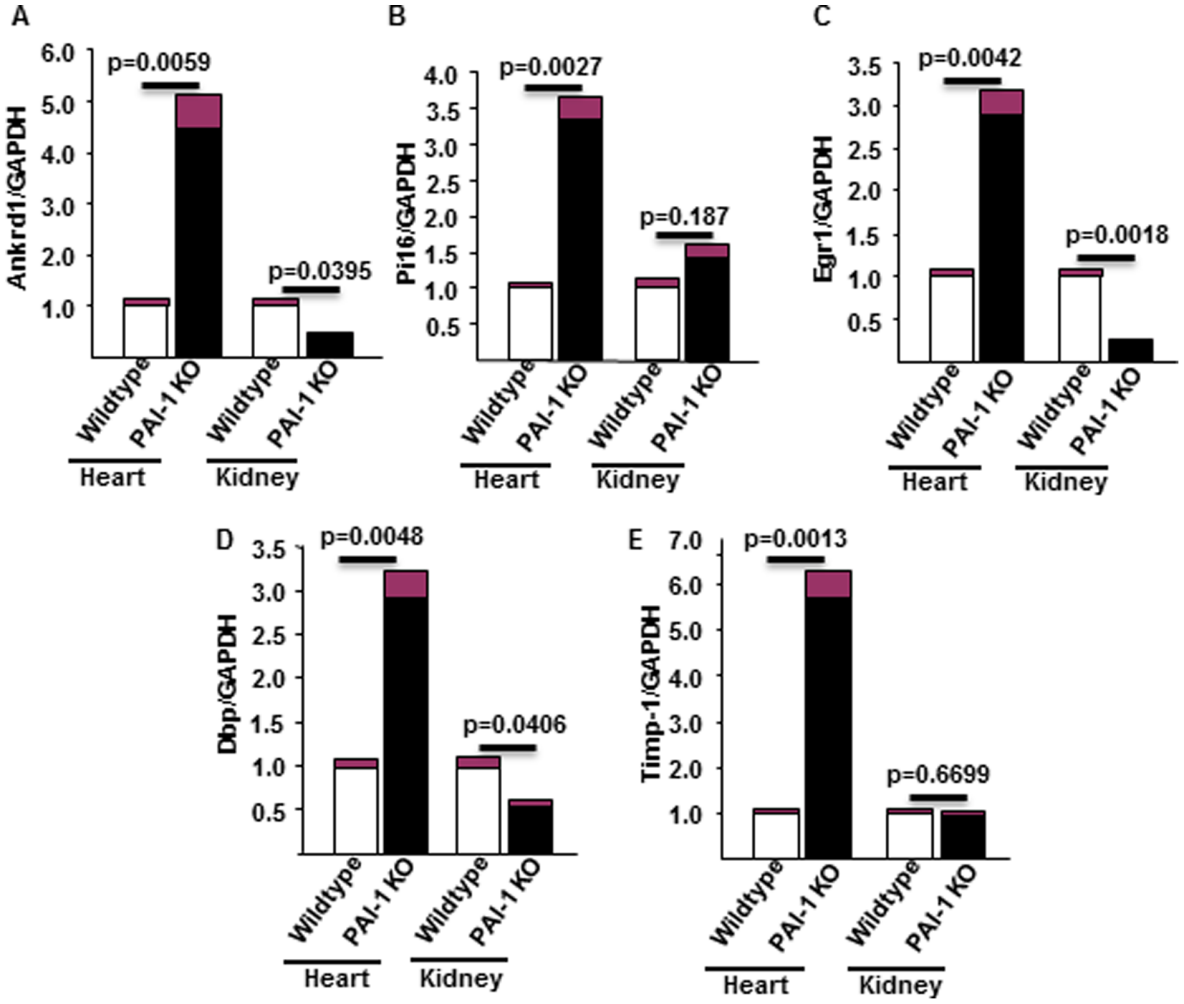
Validation of expression levels of mRNAs in aged PAI-1 knockout and wildtype hearts and kidneys by qPCR analysis. Total RNA from wildtype and PAI-1 knockout heart and kidney tissues were used for quantitation of **A.** Ankrd1; **B.** Pi16; **C.** Egr1: **D.** Dbp and **E.** Timp1 by qPCR analysis using gene specific primers. Data represents mean of triplicates ±SEM. P value of each sample was indicated in the Figure A–E.

The expression levels of five differentially expressed genes related to wound healing, hypertrophy and matrix remodeling were validated by qPCR. These are Ankrd1, Pi16, Egr1, Dbp and Timp-1 (**Figure 5A–E**). Ankrd1 is an ankyrin repeat domain containing a transcription modulator and is involved in wound healing [25], [26]. Quantitation of Ankrd1 mRNA by qPCR in wildtype and PAI-1 knockout heart and kidney samples revealed that while the level of Ankrd1 was significantly elevated in PAI-1 knockout hearts compared to wildtype controls (upregulated 4.48-fold; p = 0.005), the level of Ankrd1 mRNA was significantly decreased in PAI-1 knockout kidneys compared to wildtype kidneys (downregulated 0.45 fold; p = 0.03) (**Figure 5A** **)**. Pi16 is a serine protease inhibitor and is elevated in hypertrophic hearts [27]. qPCR analysis of mRNA revealed that the level of Pi16 mRNA was significantly elevated in PAI-1 knockout hearts compared to wildtype hearts (upregulated 3.33-fold; p = 0.002). In contrast, the change in the level of Pi16 was insignificant in PAI-1 knockout kidneys compared to wildtype controls (upregulated 1.4; p = 0.187) (**Figure 5B**). Egr1, an early growth response gene, is involved in inflammation and fibrogenesis [28]–[31]. The quantitative analysis of mRNA revealed that while the level of Egr1 was significantly elevated in PAI-1 knockout hearts compared to wildtype hearts (upregulated 2.89-fold; p = 0.004), the level of Egr1 was significantly downregulated in PAI-1 knockout kidneys compared wildtype kidneys (downregulated 4-fold; p = 0.001) (**Figure 5C**). DBP, the albumin promoter D-box binding protein, is a member of the PAR bZIP (Proline and Acidic amino acid-Rich basic leucine zipper) transcription factor family [32]. DBP is a regulator of hypoxia inducible factors (HIF-1). The level of DBP was significantly increased in PAI-1 knockout hearts compared to wildtype hearts (upregulated 2.9-fold; p = 0.004). In contrast, the level of DBP in PAI-1 deficient kidneys was significantly decreased (downregulated 2-fold; p = 0.04) (**Figure 5D**). The tissue inhibitor of matrix metalloproteinase (Timp1) controls the activity of specific matrix metalloproteinase (MMPs) and thus is involved in matrix remodeling and fibrogenesis [33]. The Timp1 mRNA level was significantly upregulated in PAI-1 knockout hearts compared to wildtype hearts (upregulated 5.7 fold; p = 0.0013). In contrast, there was no change in the expression level of Timp1 in PAI-1 knockout kidneys compared to wildtype kidneys (change 1.00 in knockout vs. 0.94 in wildtype; p = 0.66) (**Figure 5E**). Collectively, these results suggest that several genes involved in profibrotic responses are deregulated in hearts and kidneys in the absence of PAI-1.

### Effect of TGF-ß on Ankrd1, Pi16, Timp1 and Timp4 Expression in Mouse Cardiac Endothelial Cells

**Figure 6:**
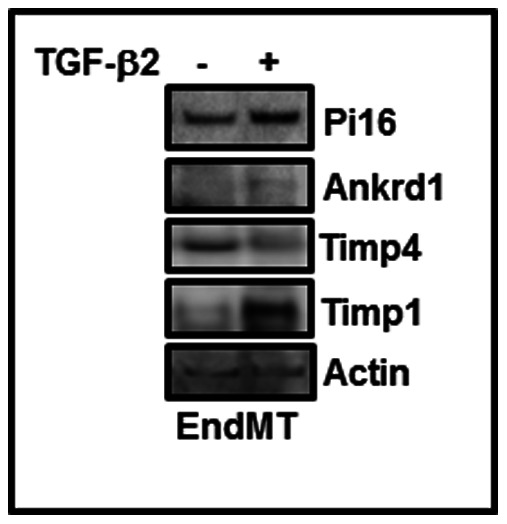
Levels of Ankrd1, Pi16, Timp4 and Timp1 in TGF-β-treated endothelial cells. Mouse cardiac endothelial cells were treated with TGF-ß2 (10 ng/ml) for 7 days. At the end of incubation, control and EndMT-derived fibroblast-like cells were harvested and whole lysates were prepared. Equal amounts of proteins were subjected to Western blot using specific antibodies as indicated.

As i) PAI-1 deficient mouse cardiac endothelial cells are more susceptible to EndMT [8], ii) EndMT-derived fibroblast-like cells synthesize excessive collagen and other profibrogenic factors compared to control cells [5], [8], [18], iii) the number of FSP-1 positive cells are significantly higher in PAI-1 knockout myocardial tissues [8], and iv) EndMT derived fibroblast-like cells in adult myocardium contributes to fibrogenesis (reviewed in [5] and references therein) here, we measured the protein levels of a few differentially expressed genes during EndMT. Equal amounts of protein from control mouse cardiac endothelial cells and TGF-ß-induced EndMT-derived cells were subjected to Western blot analysis using specific antibodies against Ankrd1, Pi16, Timp1 and Timp4. Western blot results revealed that while the levels of Pi16, Ankrd1 and Timp1 proteins were elevated in response to TGF-ß signaling, the level of Timp4 was decreased (**Figure 6**).

## Discussion

Organ fibrosis is one of the major causes of organ failure-related deaths among the millions of patients with heart failure, renal failure, idiopathic pulmonary fibrosis, liver cirrhosis and systemic sclerosis [2], [34]–[42]. Currently, there is no effective therapy for fibrosis. Although numerous studies have described the roles of different cytokines, transcriptional regulators and modulators involved in the processes of fibrogenesis [5], [6], [43], the major contributor in the initiation of fibrogenesis is uncertain. As TGF-ß is the major architect of fibrogenic pathway activation, it was initially thought that antibody-mediated suppression of TGF-ß activity would be an ideal approach to block fibrogenesis. However, blocking of TGF-ß activity would be deleterious because TGF-ß also controls other important biological processes including immunity, cellular proliferation and migration [37], [44]. Therefore, there is an urgency to identify the igniters of fibrogenesis in order to develop a novel therapeutic approach to treat fibrosis. Previous studies demonstrated that aged plasminogen activator inhibitor-1 (PAI-1) knockout mice develop cardiac-selective fibrosis without affecting any other organs [8]–[10]. Here, we asked whether profibrotic factors responsible for fibrogenesis are differentially expressed in PAI-1 knockout hearts compared to PAI-1 knockout kidneys.

In order to identify the contributor (s) of cardiac fibrosis, we examined global differential gene expression in hearts and kidneys derived from PAI-1-knockout mice and age-matched wildtype controls. The present study identified several deregulated genes in PAI-1 knockout hearts compared to wildtype hearts and knockout kidneys. Several of these deregulated genes including Ankrd1, Egr1, Pi16, Dbp and Timp1 are related to wound healing, hypertrophy and transcriptional regulation of fibrogenesis [25], [27]–[32], [45]. Most importantly, while the expression profile of many of these genes was upregulated in PAI-1 knockout hearts, the expression level of those genes was either unaffected or downregulated in PAI-1 knockout kidneys. These results indicate that these differentially expressed genes may contribute to the initiation of cardiac fibrogenesis, and PAI-1 knockout kidneys may be protected from fibrogenesis due to downregulation of these profibrotic regulators. The biology of these identified differentially expressed genes is discussed below in the light of fibrogenesis.

The present study demonstrated that Ankrd1 (Ankyrin repeat domain1) was upregulated in PAI-1 knockout hearts but not in PAI-1 knockout kidneys. Ankrd1, a 36 kDa nuclear and cytoplasmic protein, is involved in signal transduction, cytokine stimulus response and RNA Pol II mediated transcriptional regulation [45]. In response to stretch stimuli, Ankrd1 translocates to the nucleus and acts as a nuclear signaling protein upon interaction with transcription factors and thus controls their function. Ankrd1 plays a significant role in both positive and negative transcriptional regulation of cardiac gene expression as a transcriptional cofactor [45]. It has been reported that there is a left-right asymmetric distribution of Ankrd1, with increased level of expression in the left ventricle compared to right ventricle in a diastolic heart failure model [46]. Present results also demonstrated elevated levels of Ankrd1 protein in TGF-ß-induced EndMT-derived fibroblast-like cells. Thus, elevated levels of Ankrd1 in PAI-1 knockout hearts may have contributions in the onset of cardiac fibrogenesis.

The present study revealed that while the level of Egr1 was elevated in PAI-1 knockout hearts compared to wildtype hearts and PAI-1 knockout kidneys, the level of Egr1 was decreased in PAI-1 knockout kidneys compared to wildtype controls. Egr-1(Early growth response gene-1), an 82 kDa zinc finger transcription factor, is a known inducer of collagen synthesis and may contribute in the pathogenesis of fibrosis [28]–[31]. Interestingly, overexpressed Ankrd1 induces Egr-1 protein, a potent profibrotic regulator, *in vitro* [47]. Therefore, it is reasonable to propose that the presence of excess Ankrd1 in aged PAI-1 knockout hearts may be one of the key igniters of fibrogenesis via activation of the potent profibrotic transcription factor, Egr1. In contrast, PAI-1 knockout kidneys are protected from the onset of fibrogenesis due to lack of Ankrd1-Egr1 profibrogenic axis.

The expression level of Pi16 (peptidase inhibitor 16), a 47 kDa peptidase inhibitor, was significantly elevated in PAI-1 knockout hearts. In contrast there was no alteration in Pi16 expression in PAI-1 knockout kidneys, indicating influence of PAI-1 deficiency on Pi16 expression is organ and tissue-specific. Pi16 is a known regulator of cardiac hypertrophy and is upregulated in hypertrophy and heart failure. Although, cardiomyocyte-specific Pi16 overexpressing transgenic mice showed normal cardiac function, the size of their hearts were smaller with hypotrophic cardiomyocytes [27] indicating that Pi16 may be anti-hypertrophic. At present, the exact role of elevated Pi16 in cardiac fibrogenesis and the associated downstream pathway in PAI-1 knockout hearts is unknown. The present data also showed that the protein level of Pi16 was elevated during EndMT in response to TGF-ß2, indicating Pi16 is a target of TGF-ß signaling. Further characterization of Pi16 in profibrotic signaling and its contribution to collagen and other matrix protein synthesis *in vitro* is required.

The DBP (albumin D-site binding protein), a 34 kDa PAR-bZIP transcription factor, was upregulated in PAI-1 knockout hearts and downregulated in knockout kidneys compared to wildtype controls. Dbp is known to bind to promoters of many genes and control the expression levels of those genes, such as E-box driven clock genes CYP2A4 and CYP2A5 [32]. DBP also directly interacts with a novel promoter within the first exon of human PPAR-γ gene and activates its circadian expression [48]. At present, the biological significance of altered expression of DBP in PAI-1 knockout hearts and kidneys is not clear. As DBP is involved in a number of biological processes such as cell differentiation, proliferation and apoptosis [32], it will be interesting to study whether elevated DBP plays any role in profibrotic signaling and cardiac fibrogenesis.

The present study also identified a number of genes, involved in matrix remodeling under pathological conditions that were uniquely upregulated in PAI-1 knockout hearts compared to PAI-1 knockout kidneys but not in the wildtype tissues. These included Scx, IGFBP6, Klf6, Notch4, Egr1, Timp1, Timp2 and MMP23. i) Scx (Scleraxis), a 22 kDa basic-helix-loop helix transcription factor, is an important regulator of collagen gene expression and plays a significant role in matrix remodeling and fibrosis. Importantly, the level of Scx is significantly elevated in fibrotic tissues [3], [49]. Therefore, elevated levels of Scx in PAI-1 knockout hearts may contribute to cardiac fibrogenesis; ii) IGFBP6 (Insulin like growth factor binding protein 6), a 29 kDa modulator of IGF activity, induces cellular proliferation, suppresses apoptosis and delays cellular senescence. IGFBP6 may be involved in liver fibrogenesis [50], [51] indicating elevated IGFBP6 in PAI-1 knockout hearts may play a role in cardiac-selective fibrogenesis; iii) Klf6, a 31 kDa zinc finger transcription factor, regulates TGF-β gene expression and is associated with fibrosis in non-alcoholic fatty liver diseases. Klf6 is also involved in oxidative stress-induced stellate cell activation, inflammation and increased collagen synthesis [52]–[54]. Therefore, elevated level of Klf6 may play a role in cardiac-specific fibrosis; iv) Notch4, a 230 kDa Notch signaling receptor, is significantly elevated in heart failure and may play a role in myocardial matrix remodeling [55], indicating elevated levels of Notch4 in PAI-1 knockout hearts may have a positive role in cardiac fibrogenesis; v) Egr1, a target of TGF-ß-induced ERK1/2 MAP kinase signaling, plays a significant role in fibrogenesis [28]–[31]. The elevated level of Egr1 in PAI-1 knockout hearts compared to PAI-1 knockout kidneys illustrates its role in cardiac fibrogenesis as discussed earlier; vii) Timp1 and Timp 2 (23 kDa and 21 kDa tissue inhibitor of metalloproteinase 1 and 2) control the activity of metalloproteinases (MMPs) and are involved in matrix remodeling. The levels of Timp1 and Timp2 are elevated in fibrotic tissues and involved in fibrogenesis [56]; Thus, Timps and MMPs are biomarkers for physiological and pathological matrix remodeling [33], [57]. The present study revealed that the levels of Timp1 and Timp2 were significantly elevated in PAI-1 knockout hearts compared to age-matched wildtype controls and knockout kidneys. In contrast, there was no change in expression of Timp1 and Timp2 in PAI-1 knockout kidneys compared to wildtype kidneys suggesting Timp1 and Timp2 may play a significant role in the initial phase of cardiac matrix remodeling in aged PAI-1 knockout mice via controlling MMP activities and matrix degradation; vii) MMP23, a 44 kDa metalloproteinase, may be involved in matrix remodeling under pathological conditions and the level of MMP23 is significantly elevated in idiopathic pulmonary fibrosis (IPF) [58], [59]. As the level of MMP23 was significantly elevated in PAI-1 knockout hearts compared to knockout kidneys, MMP23 may contribute in cardiac fibrogenesis; and viii) Loxl1 (lysyl oxidase like 1 protein), a 53 kDa lysyl oxidase like protein, catalyses the formation of crosslinks in collagen and other extracellular matrix proteins and stabilizes the protein structure. The deregulation of Loxl1 is associated with different tissue fibrosis [60], [61]. The present study identified Loxl1 as an upregulated gene in PAI-1 knockot hearts compared to knockout kidneys, thus indicating its possible role in cardiac fibrosis. Collectively, these results indicate that along with elevated Ankrd1-Egr1 axis, a subset of upregulated regulators involved in profibrogenic signaling and matrix remodeling may contribute to cardiac-specific fibrosis in PAI-1 knockout mice.

**Figure 7:**
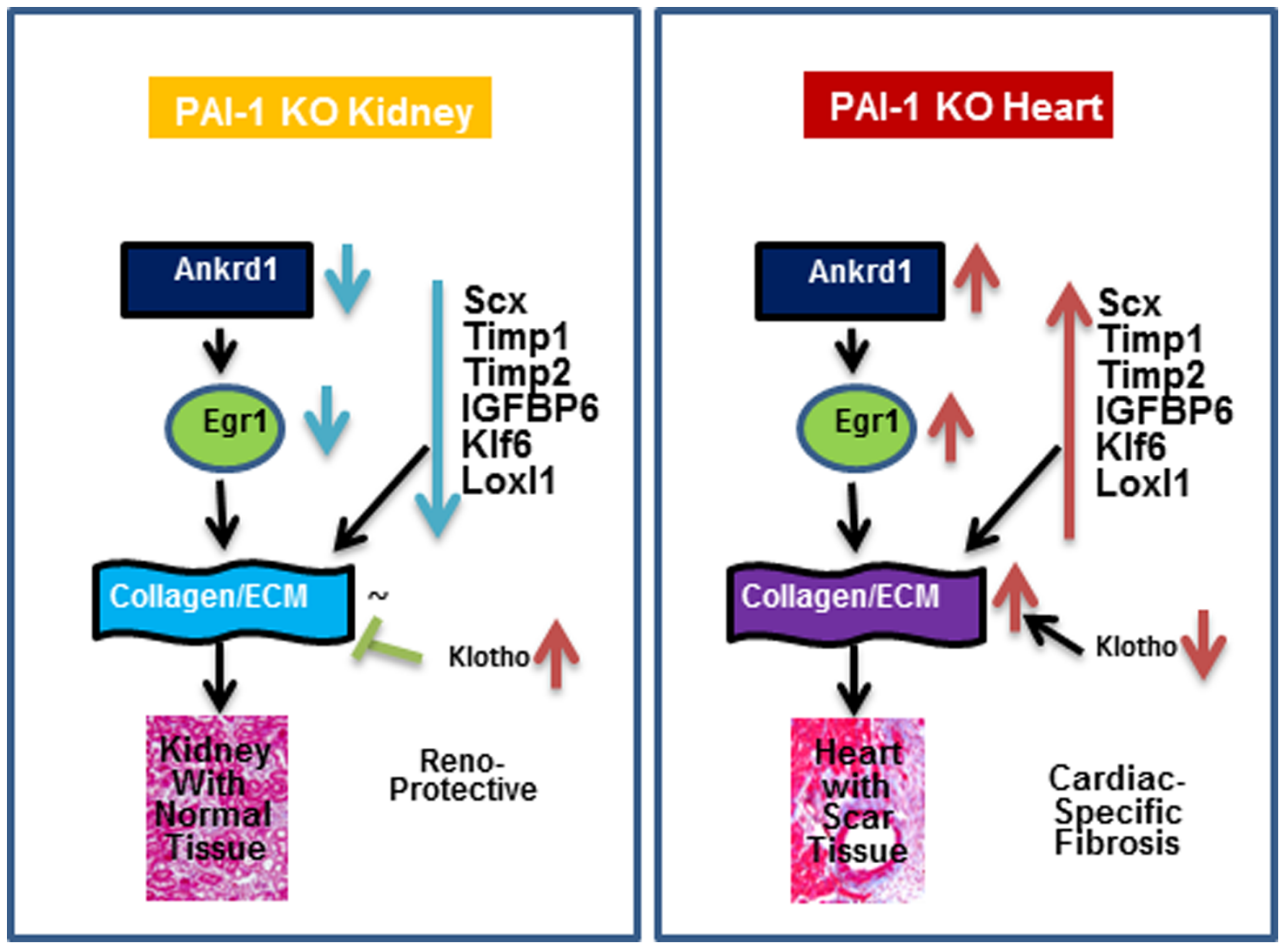
Schematic diagram showing possible involvement of Ankrd1-Egr1 axis and a subset of profibrotic factors as igniters of cardiac fibrosis. Along with other key profibrogenic factors, Ankrd1-Egr1 axis may play a pivotal role in initiation of cardiac-selective fibrosis; and kidney is protected from PAI-1 deficiency-induced fibrogenesis due to lack of these profibrotic regulators. Antifibrotic Klotho may also protect kidney from fibrogenesis.

Additionally, the level of expression of Klotho (Kl) in PAI-1 knockout kidneys was significantly higher (20-fold) compared to knockout hearts (Table S3). A recent report demonstrates that Klotho blocks TGF-ß-induced profibrotic signaling by direct interaction with TGF-ß RII and thus ameliorates renal fibrosis [62]. Furthermore, low level of Klotho in the kidney is associated with worse renal interstitial fibrosis in the unilateral ureteral obstruction (UUO) mouse model [63]. As Klotho is a known antifibrotic factor, we cannot rule out the possibility of Klotho-mediated protection of PAI-1 knockout kidneys from fibrogenesis. Therefore, in contrast to upregulation of a subset of profibrotic factors and downregulation of antifibrotic factors in PAI-1 knockout hearts that lead to fibrogenesis, downregulation of those profibrotic factors and upregulation of antifibrotic factor in PAI-1 knockout kidneys may protect it from fibrogenesis (**Figure 7**).

Other than pro- and antifibrotic genes, numerous genes whose products are involved in a wide variety of biological processes such as adhesion, proliferation, migration, inflammation, apoptosis and metabolism, were also differentially expressed in PAI-1 knockout hearts and kidneys. Therefore, we cannot rule out the possibility of involvement of these genes in the initiation of cardiac-specific fibrosis and in the prevention of renal fibrosis. The rationale behind this notion comes from the observations that fibrogenic pathways can be activated or attenuated by different metabolic end products [64]; activation or differentiation of myofibroblasts from fibroblasts, endothelial cells or epithelial cells depends on other biological processes of these cells such as adhesion, apoptosis and senescence [65]. Taken together, the results of the present study on global gene expression profiling in PAI-1 knockout hearts and kidneys established a strong functional link of PAI-1 with different tissue-specific biological processes.

Although the primary goal of this study was the identification of possible profibrogenic factors differentially expressed in PAI-1 knockout hearts and kidneys, an added benefit is the massive data on global gene expression profiling and the involvement of differentially expressed genes in a variety of biological processes in hearts and kidneys now available (online supplemental data) to other researchers working in the field of plasminogen-plasmin/u-PA/tPA/PAI-1 biology and PAI-1-associated diseases [5]. While many differentially expressed genes in PAI-1 knockout hearts and kidneys are simply due to tissue-specific gene expression, several genes involved in a number of biological processes are influenced by PAI-1 deficiency. To avoid tissue-specific differential expression of genes in the heart vs. kidney comparison, we further filtered and identified the genes which are uniquely expressed only in the PAI-1 knockout heart vs. PAI-1 knockout kidney comparison, and not common in the wildtype heart and kidney comparison vs. PAI-1 knockout heart and kidney comparison. However, there are several limitations dealing with global gene expression profiling using myocardial and renal tissues. One such limitation is the use of total RNA extracted from tissues. When using a mixture of RNA from different cell types including endothelial cells, fibroblasts, epithelial cells and myocytes, it is impossible to predict the specific cell type that is contributing to the differential expression of a particular gene. A second limitation is that activities of numerous transcription factors or regulators, controlling different biological processes in response to stress or signals, are altered only at the posttranslational levels like phosphorylation, acetylation etc. Therefore, it is another hurdle to identify those altered regulators in PAI-1 knockout hearts or kidneys by global gene expression profiling. For example, the levels of phosphorylated Smad2/3 are significantly elevated in PAI-1 knockout hearts compared to wildtype hearts [8]. Despite these limitations, the differential expression profile of each gene presented in this study came from whole tissue, a natural environment where expression patterns of a particular gene by a specific cell-type may be influenced by secreted products of other cell types as described [66].

In summary, the present study on global gene expression profiling of PAI-1 knockout and wildtype hearts and kidneys helps us to formulate a possible and robust hypothesis defining a pathway involving Ankrd1-Egr1-ECM genes that may explain the molecular basis of cardiac-selective fibrosis and lack of renal fibrosis in aged PAI-1 knockout mice. Along with the Ankrd1-Egr1 axis, a subset of profibrotic genes including Scx, Timp1/2, IGFBP6, Klf6 and Loxl1 may contribute to cardiac fibrogenesis. These genes were uniquely upregulated in PAI-1 knockout hearts compared to PAI-1 knockout kidneys (**Figure 7**). Therefore, the present study has established a platform for the future study of organ-specific fibrosis, the identification of igniters of fibrogenesis and the design of novel therapeutic approaches that could prevent either the onset of post-injury fibrogenesis or progression of initiated fibrosis.

## Supporting Information

Figure S1 **Collagen deposition in aged wildtype and PAI-1 knockout lungs and livers is comparable.** The levels of collagen accumulation in lungs and livers derived from 24 m old wildtype and PAI-1 knockout mice were determined by Masson’s trichrome staining. **A,B.** Livers from 24 m old wildtype mouse (**A**) and 24 m old PAI-1 knockout mouse (**B**). **C,D.** Lungs from 24 m old wildtype mouse (**C**) and 24 m old PAI-1 knockout mouse (**D**).Quantitative data on collagen deposition are presented in the text.
(TIF)

Table S1 **Differential gene expression in wildtype and PAI-1 knockout hearts.** Up and Down regulation. [(+) downregulated genes in KO hearts compared to WT hearts; (−) upregulated in KO hearts compared to WT hearts]
(XLS)

Table S2 **Differential gene expression in wildtype and PAI-1 knockout Kidney.** Up and Down regulation. [(+) downregulated genes in KO kidneys compared to WT kidneys; (−) upregulated in KO kidneys compared to WT kidneys]
(XLS)

Table S3 **Differential gene expression in PAI-1 knockout hearts and kidneys.** Up and Down regulation: Common. [(+) upregulated genes in KO hearts compared to KO kidneys; (−) downregulated in KO hearts compared to KO kidneys]
(XLS)

Table S4 **Differential gene expression in wildtype hearts and kidneys.** Up and Down regulation: Common. [(+) upregulated genes in WT hearts compared to WT kidneys; (−) downregulated in WT hearts compared to WT kidneys]
(XLS)

Table S5 **Differential gene expression in PAI-1 knockout hearts and kidneys.** Up and Down regulation: Unique. [(+) upregulated genes in KO hearts compared to KO kidneys; (−) downregulated in KO hearts compared to KO kidneys]
(XLS)

Table S6 **Differential gene expression in wildtype hearts and kidneys.** Up and Down regulation: Unique. [(+) upregulated genes in WT hearts compared to WT kidneys; (−) downregulated in WT hearts compared to WT kidneys]
(XLS)
