# Supplementary figures and images for: Global Gene Expression Profiling in PAI-1 Knockout Murine Heart and Kidney: Molecular Basis of Cardiac-Selective Fibrosis
Source: PLoS One. 2013 May 28;8(5):e63825. doi: 10.1371/journal.pone.0063825 (PMC3665822; doi:10.1371/journal.pone.0063825)

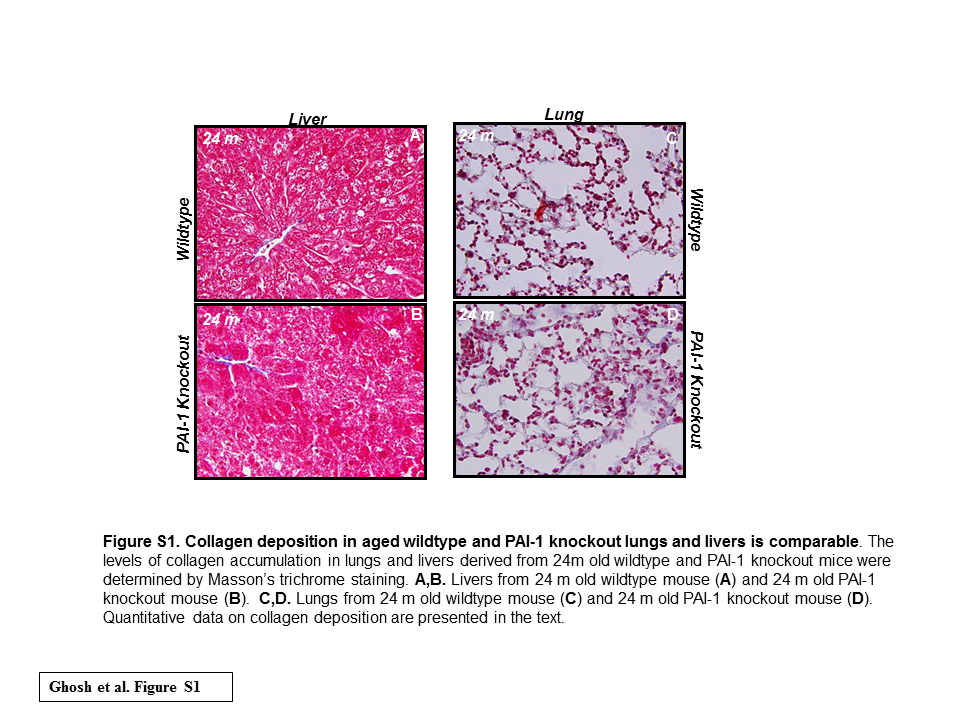

Supplement: Figure S1 — Collagen deposition in aged wildtype and PAI-1 knockout lungs and livers is comparable. The levels of collagen accumulation in lungs and livers derived from 24 m old wildtype and PAI-1 knockout mice were determined by Masson’s trichrome staining. A,B. Livers from 24 m old wildtype mouse (A) and 24 m old PAI-1 knockout mouse (B). C,D. Lungs from 24 m old wildtype mouse (C) and 24 m old PAI-1 knockout mouse (D).Quantitative data on collagen deposition are presented in the text. (TIF) [file pone.0063825.s001.tif]
